# Supplementary material for: Oligomerization Affects the Ability of Human Cyclase-Associated Proteins 1 and 2 to Promote Actin Severing by Cofilins
Source: Int J Mol Sci. 2019 Nov 12;20(22):5647. doi: 10.3390/ijms20225647 (PMC6888645; doi:10.3390/ijms20225647)
Supplement: Supplementary file 1 [file ijms-20-05647-s001.pdf]

Supplementary Information for

Oligomerization Affects the Ability of Human Cyclase-Associated Proteins 1 and 2 to Promote Actin Severing by Cofilins

Vedud Purde, Florian Busch, Elena Kudryashova, Vicki H. Wysocki, and Dmitri S. Kudryashov

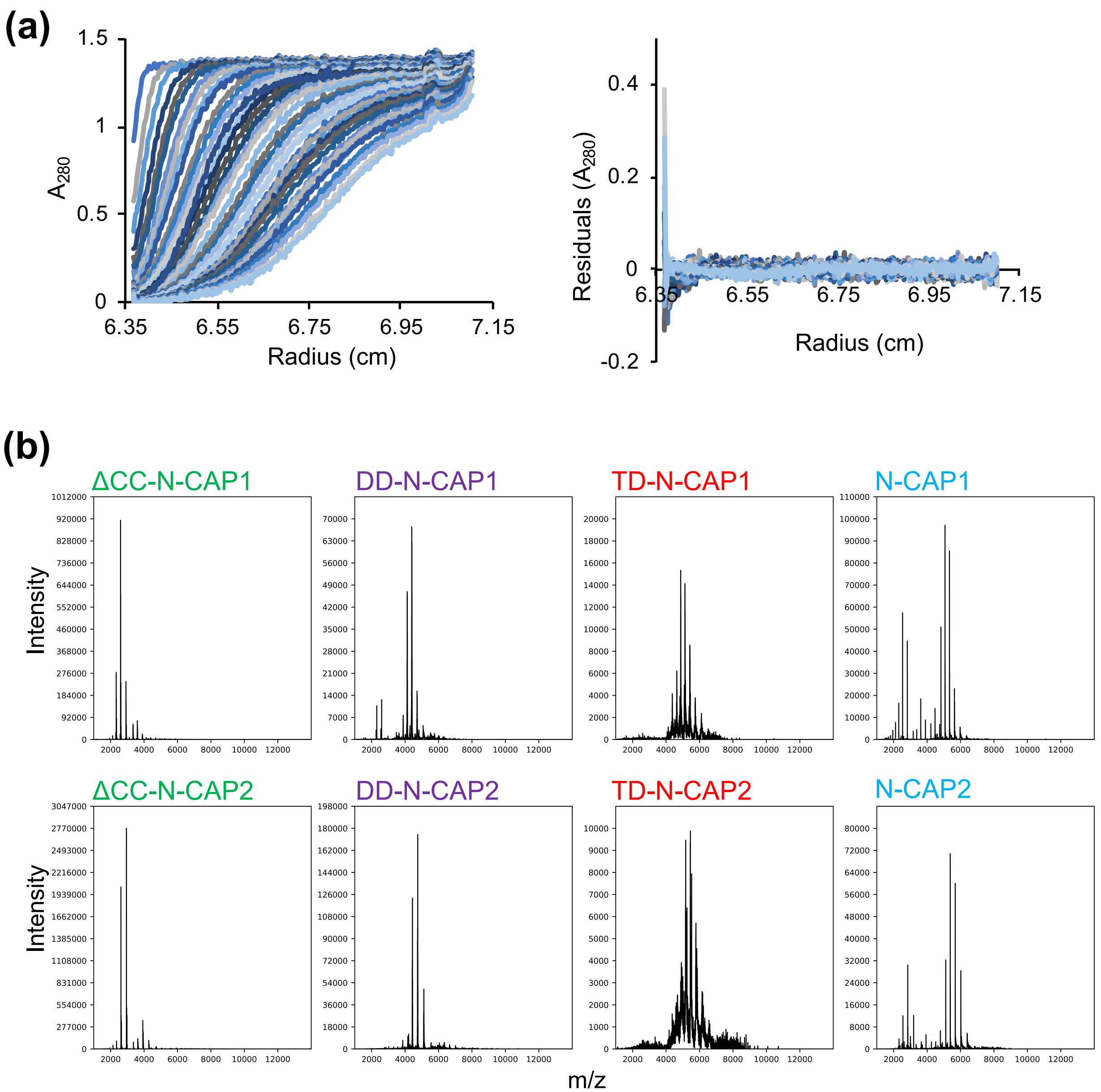

Supplementary Figure S1. Radial signal profiles of SV-AUC and raw m/z data from native MS experiments. Related to Figure 2.

(**a**) Representative example of the radial signal profiles of the sedimentation velocity analytical ultracentrifugation (SV-AUC) experiment. Sedimentation profile data for ΔCC-N-CAP1 fitted using SEDFIT software and fit residuals are shown. (**b**) Raw m/z data from native MS analysis.

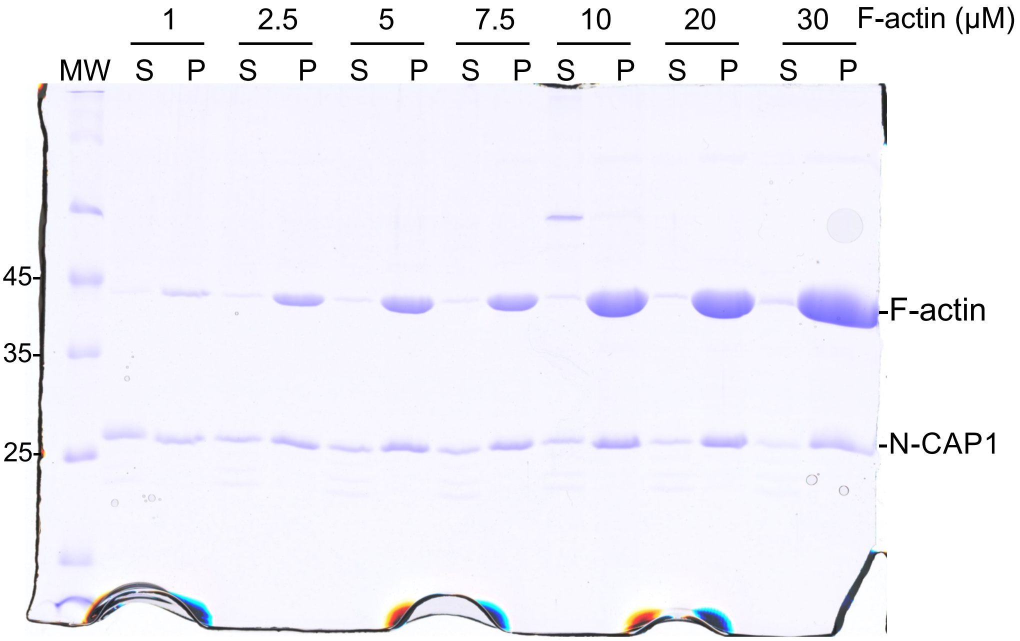

Supplementary Figure S2. Uncropped version of the gel shown in Figure 4a.

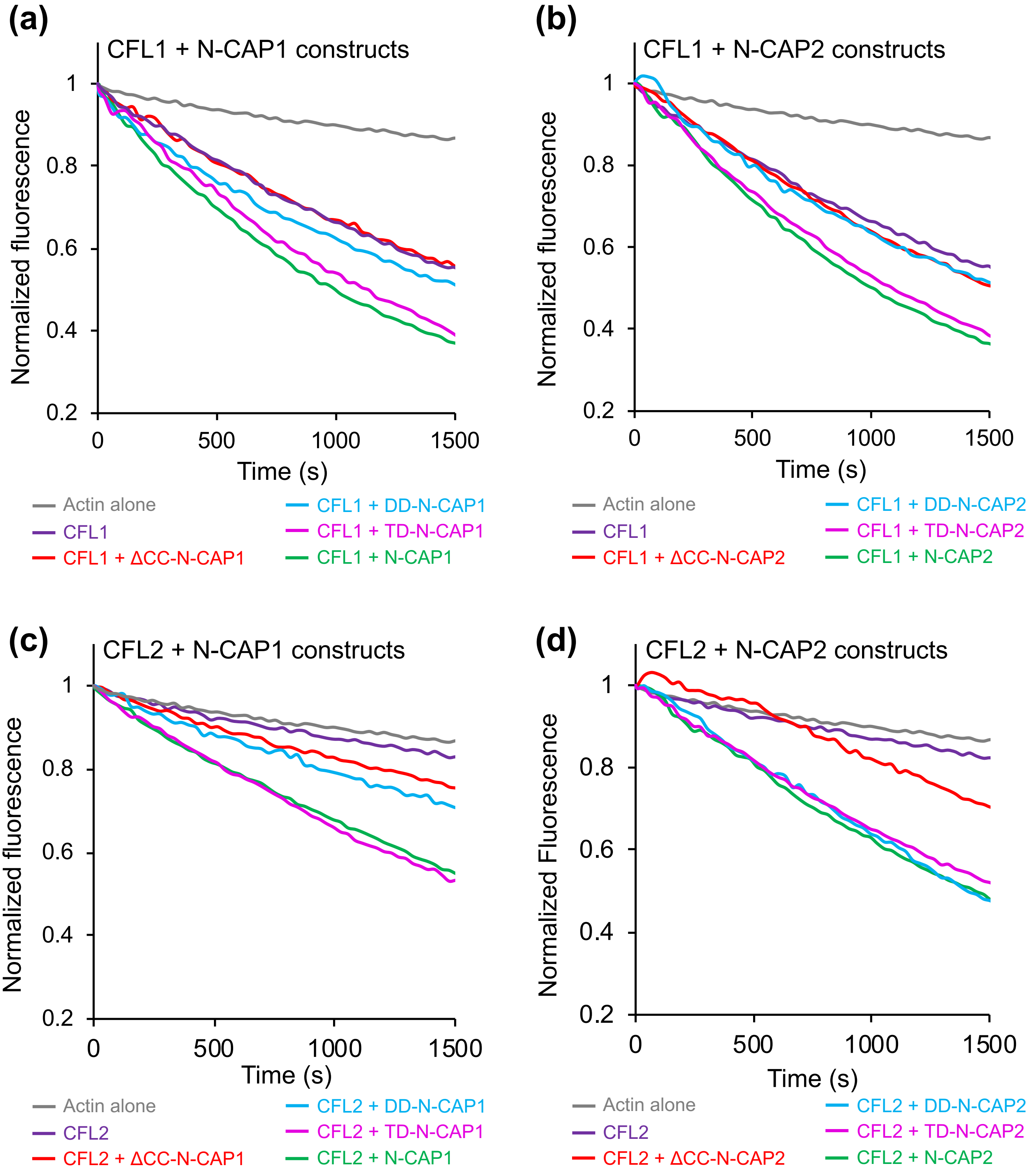

Supplementary Figure S3. Effects of N-CAP oligomers on cofilin-mediated F-actin disassembly in bulk pyrene–actin depolymerization assays. Related to Figure 5.

(**a-d**) Traces represent means (n=3) of pyrene-actin fluorescence decay profiles monitored upon depolymerization of F-actin in the presence of CFL1 (**a**,**b**) or CFL2 (**c**,**d**) and N-CAP1 (**a**,**c**) or N-CAP2 (**b**,**d**) oligomeric constructs of different stoichiometry: monomers (ΔCC-N-CAP1, ΔCC-N-CAP2), dimers (DD-N-CAP1 and DD-N-CAP2), trimers (TD-N-CAP1 and TD-N-CAP2) and tetramers (N-CAP1 and N-CAP2) as indicated below each graph.

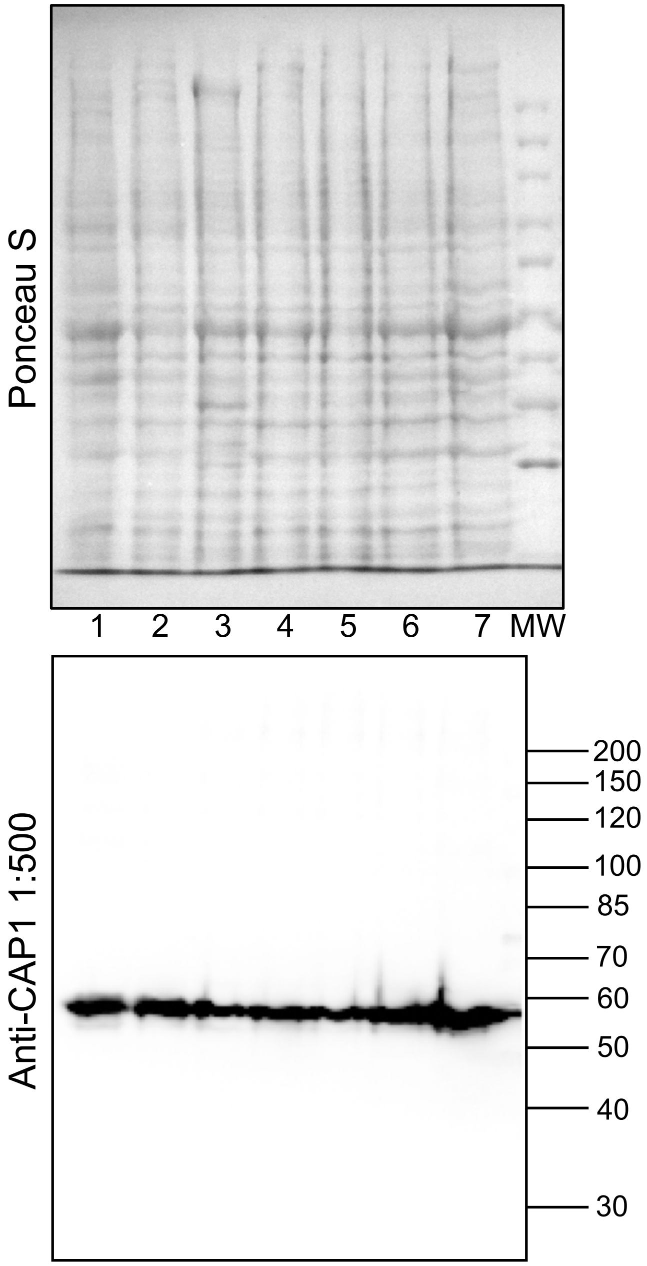

Supplementary Figure S4. Western blot analysis of whole cell lysates (WCL) using anti-CAP1 (Sigma #SAB1406999). Uncropped version of the anti-CAP1 blot (Figure 7a). Western blot lanes numbers are as followed (50 μg of WCL per well):

1 – Hs 578T;
2 – HT-1080;
3 – SKBR-3;
4 – MDA-MB-436;
5 – HeLa;
6 – U2OS;
7 – WI-38;
MW – protein molecular weight ladder.

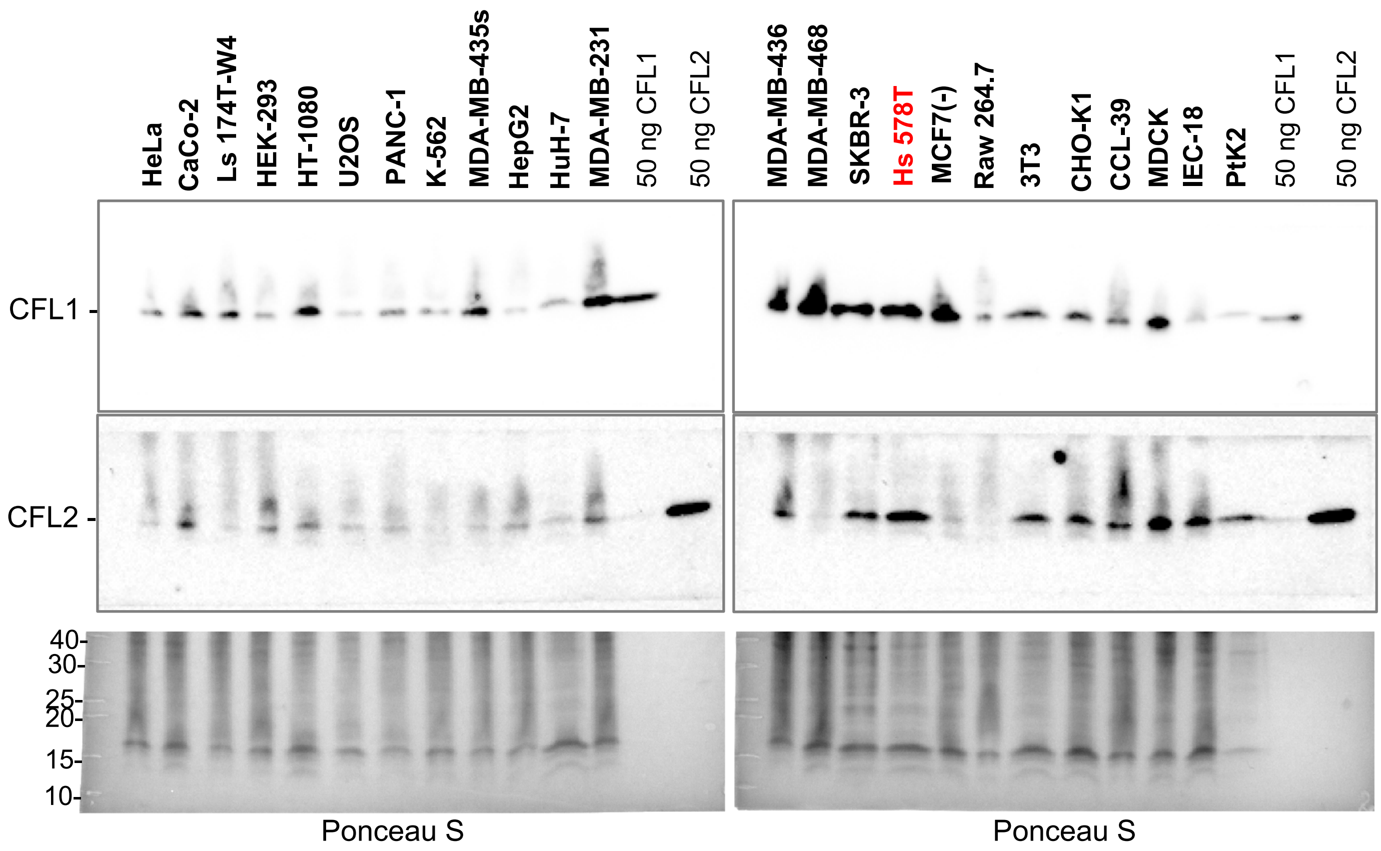

Supplementary Figure S5. Western blot analysis of whole cell lysates (WCL) using isoform-specific anti-cofilin antibodies (rabbit anti-CFL1 (Cell Signaling Technology #5175) and rabbit anti-CFL2 (Abgent #AP20625c)). Uncropped versions of the anti-CFL1 and anti-CFL2 blots (Figure 7a).

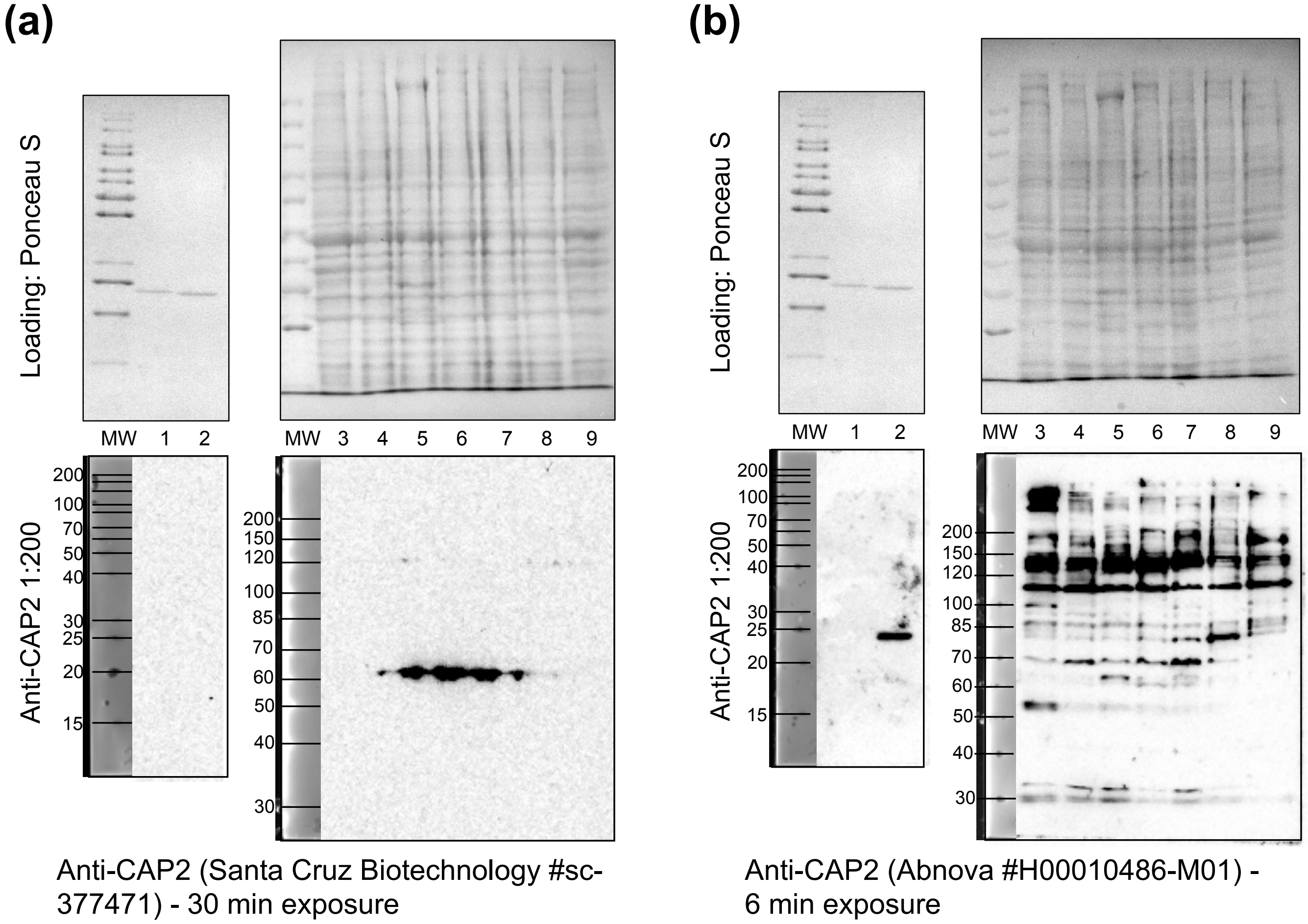

Supplementary Figure S6. Two tested commercially available anti-CAP2 antibodies of mouse origin are unsuitable for PLA application as revealed by western blot analysis of recombinant proteins and whole cell lysates (WCL). Related to Figure 7.

Western blot lanes (MW – protein molecular weight ladder):

1 – recombinant human CAP1 fragment (30-220 a.a.; ΔCC-N-CAP1), 50 ng (MW=23kDa);

2 – recombinant human CAP2 fragment (30-220 a.a.; ΔCC-N-CAP2), 50 ng (MW=23kDa);

3-9 – WCL, 50 μg/well: 3 – Hs 578T, 4 – HT-1080, 5 – SKBR3, 6 – MDA-MB-436, 7 – HeLa, 8 – U2OS, 9 – WI-38.

(**a**) Using anti-CAP2 from Santa Cruz Biotechnology (#sc-377471) raised against amino acids 77-121 of human CAP2, at a long exposure time, a band of ~60kDa appears in some WCL, but it is most likely non-specific (e.g., related to residual immunoglobulin from FBS), since this antibody fails to recognize the recombinant human ΔCC-N-CAP2 (30-220 a.a.).

(**b**) While anti-CAP2 from Abnova (#H00010486-M01) raised against GST-tagged full-length recombinant human CAP2 specifically recognizes the recombinant human ΔCC-N-CAP2 (30-220 a.a.) and not the ΔCC-N-CAP1, staining of WCL produces multiple major non-specific bands, which makes this antibody unusable for PLA.
